# Biosensor Technologies for Early Detection and Quantification of Plant Pathogens

**DOI:** 10.3389/fchem.2021.636245

**Published:** 2021-06-02

**Authors:** Kazbek Dyussembayev, Prabhakaran Sambasivam, Ido Bar, Jeremy C. Brownlie, Muhammad J. A. Shiddiky, Rebecca Ford

**Affiliations:** ^1^ Centre for Planetary Health and Food Security, Griffith University, Nathan, QLD, Australia; ^2^ School of Environment and Science, Griffith University, Nathan, QLD, Australia; ^3^ Queensland Micro and Nanotechnology Centre, Griffith University, Nathan, QLD, Australia

**Keywords:** plant disease, plant pathogen detection, rapid diagnostics, biosensor, nanotechnology

## Abstract

Plant pathogens are a major reason of reduced crop productivity and may lead to a shortage of food for both human and animal consumption. Although chemical control remains the main method to reduce foliar fungal disease incidence, frequent use can lead to loss of susceptibility in the fungal population. Furthermore, over-spraying can cause environmental contamination and poses a heavy financial burden on growers. To prevent or control disease epidemics, it is important for growers to be able to detect causal pathogen accurately, sensitively, and rapidly, so that the best practice disease management strategies can be chosen and enacted. To reach this goal, many culture-dependent, biochemical, and molecular methods have been developed for plant pathogen detection. However, these methods lack accuracy, specificity, reliability, and rapidity, and they are generally not suitable for *in-situ* analysis. Accordingly, there is strong interest in developing biosensing systems for early and accurate pathogen detection. There is also great scope to translate innovative nanoparticle-based biosensor approaches developed initially for human disease diagnostics for early detection of plant disease-causing pathogens. In this review, we compare conventional methods used in plant disease diagnostics with new sensing technologies in particular with deeper focus on electrochemical and optical biosensors that may be applied for plant pathogen detection and management. In addition, we discuss challenges facing biosensors and new capability the technology provides to informing disease management strategies.

## Introduction

A myriad of plant pathogens directly affects crop quality and decreases food supply, with the greatest impact occurring when the affected crop is a staple food of a large population, in a poorly resourced developing region. As evidenced throughout history, a single staple crop pathogen can cause mass starvation and/or migration (Fletcher et al., 2006). More recently, the Ug99 strain of *Puccinia graminis tritici* has posed a significant risk to global wheat production for the last three decades, most immediately in South Asia (Flood, 2010). This was of a particular concern since 90% of the analyzed wheat varieties that were initially tested were highly susceptible (Singh et al., 2011). Despite a massive effort to breed for resistance and to develop disease management strategies, an estimated 10–16% global annual productivity loss remains due to plant pathogens (Chakraborty and Newton, 2011).

Plant diseases are managed by following specific cultivation and chemical application practices including crop rotation, clean seed, resistant variety use, and seed and foliar fungicidal treatment (Sharma et al., 2011; Mancini and Romanazzi, 2014). Fungicides in particular are used to achieve an acceptable level of disease control (Davidson and Kimber, 2007). However, the long term or over use of fungicides has led to a selective rise in insensitivity within the fungal populations to certain modes of action (Donoso and Valenzuela, 2018). It is therefore, very important to devise tools to aid in the accurate, sensitive, and the speedy detection of causal pathogens for more effective pre-emptive application of optimal chemistries. This in turn will reduce unnecessary numbers and quantities of chemical applications in the cropping system, and thereby reduce the selective pressure on the pathogen populations and the potential secondary environmental impacts.

## Diagnostic Methods for Plant Pathogens

Detecting plant pathogens before disease symptoms are visible is crucial in monitoring plant health and enacting effective informed disease management (IDM) strategy. Since many fungal pathogens cause similar modifications in plants during disease development, it is essential to differentiate between causal species. More visible symptoms often occur in vulnerable crops resulting in changes in morphology and coloration, specific necrotic spots, and even the death of the plant’s stem or leaves. However, knowledge of latent infection with no visible symptoms is also crucial to understand, to ensure fully informed management (Oerke, 2020).

The earliest traditional method, still broadly used for disease and potentially pathogen diagnosis, is visual crop inspection, requiring an experienced grower or pathologist. However, it is likely that the pathogen will have become well established in host populations by the time a visual diagnosis is made. As a consequence, recent efforts have focused on the development of earlier pathogen detection methods with greater sensitivity, accuracy, and identification speed. To date, these have comprised of three types of molecular assays, which are protein-based or nucleic acid technologies: Enzyme-linked immunosorbent assay (ELISA), polymerase chain reaction (PCR) assay and loop mediated isothermal amplification (LAMP) assay.

Antibodies designed to recognize target-specific antigens, to detect and to a lesser extent, quantify levels of plant pathogens have been produced. By far the most developed serology-based diagnostics method for fungal pathogens is ELISA. This enables pathogen detection via a colorimetric reaction, visualized by the naked eye or optical reader for quantification (Fang and Ramasamy, 2015). Colorimetric reactions with monoclonal antibodies has been used to detect Botrytis fungal species that cause Botrytis Gray Mold (Meyer et al., 2000; Dewey and Yohalem, 2007). Despite the fact that traditional ELISA has become as the golden standard in the detection of almost all types of pathogens whether in environmental, chemical, biotechnological, health and agricultural analysis, it still has relatively low sensitivity and accuracy. To solve this certain problem, many scientific research have been conducted especially last decade, emphasizing the application of nanomaterials to design more improved ELISA. Nanoparticles could replace enzyme, the main component of ELISA which acts as signal label and catalyst. In other word, nanomaterials such as graphene nanosheets, polystyrene particles, and SiO_2_ nanoparticles are able to act as carriers to load fully sufficient enzymes for signal amplification and also their enzyme-like activity can catalyze the TMB/H_2_O_2_ chromogenic system which could tackle enzymes’ natural inconveniencies such as low resistance to acid and alkali, hard structure to be separated and difficulty to modify. These modifications developed the sensitivity greatly. Even though, the integration of nanomaterials and traditional ELISA has achieved significant enhancements, it is only few parts of this technique which could be modified, not as a whole improvement. Because many synthesized nanomaterials are coated with the exceeded dense surfactant on the surface that may bring to obstacles to modify antibody, some challenges still remain in the application of current ELISA-based method for pathogen detection (Wu et al., 2019; Wu et al., 2020). In addition, the design and production of antibodies is complex, resource-intensive and the assays are unsuitable for multiplexing.

For improved specificity and sensitivity of detection, alternative approaches targeting nucleic acid sequences of target pathogens were developed. These often rely on PCR amplification of the target sequence, followed by amplicon detection and visualization. A wide range of PCR amplification assay variants have been implemented in pathogen diagnostics applications to improve the sensitivity (nested and cooperation-PCR), signal detection (magnetic capture-hybridization and ELISA-PCR) and multiplexing capacity (multiplex-PCR) of the assay (Capote et al., 2012). PCR-based probes are often targeted at the ribosomal intergenic spacer or internal transcribed spacer regions (Suarez et al., 2005), or house-keeping genes such as β-tubulin (Brouwer et al., 2003; Spotts et al., 2008), cutinase A (Gachon and Saindrenan, 2004), or RNA helicase (Celik et al., 2009). PCR methods are simple, reliable, scalable, and detect a single picogram of pure fungal DNA (Nielsen et al., 2002). Increased detection sensitivities have been achieved with quantitative PCR (qPCR), which utilizes real-time monitoring of amplification-dependent fluorescence to accurately determine initial template amounts. However, this requires expensive equipment and reagents which limits its use as a rapid cost-effective diagnostic method. In addition, the high sensitivity of the assay requires normalization steps to guarantee accuracy of results (Wong and Medrano, 2005; Nowrouzian et al., 2009). Sensitivity may also be substantially reduced when detecting in a plant material background.

To improve portability of molecular diagnostic assays, the DNA amplification technique of LAMP was developed, which rapidly amplifies nucleic acids with high specificity and sensitivity under isothermal conditions (Notomi et al., 2000). LAMP-diagnostic assays were initially focused on detecting bacterial and viral pathogens (Obura et al., 2011; Bühlmann et al., 2013) and more recently on plant fungal pathogens (Tomlinson et al., 2013; Chen et al., 2016; Manjunatha et al., 2018). Chen et al. (2005) developed a rapid LAMP assay for the detection of a major fungal pathogen of chickpea, *Ascochyta rabiei*, from seed, plant debris and soil. This was based on primers targeting the ITS region and achieved a minimum detectable threshold concentration of 6.01e-6 ng/µL (Chen et al., 2016). Although the LAMP isothermal method has great advantages for plant pathogen detection, drawbacks include a reduced ease of optimal primer design compared to traditional PCR (Lau and Botella, 2017). In addition, LAMP amplicons contain a mixture of stem-loop DNA molecules of different sizes, which are not suitable for subsequent gene cloning. Meanwhile, although PCR-based assays have generally improved the sensitivity of detection and have been used for multiplexed pathogen detection (Price et al., 2010; Araujo et al., 2012; Dai et al., 2013), the assays are prone to non-specific DNA amplification resulting in false positives. This is of particular concern when performing multiplex detection on unknown pathogens in diseased plant tissues (Markoulatos et al., 2002; Sint et al., 2012).

Despite the improvements in sensitivity and specificity to particular target pathogen, these wide spread methods perform some disadvantages, such as long diagnostic time, complex sample preparation steps, or carrying the sample from field to specialized laboratories and the need for trained professionals. Accordingly, a faster, more reliable, extremely sensitive, accurate, and *in-field* diagnostic method is required. Such a “point-of-care technology” may be developed relying on the main characteristics of optics or electrochemistry. This approach creates certain enhancements during bioassays providing the short response time without the user’s skill to interpret the data and on-site experiments at low cost (Cassedy et al., 2020).

## Biosensor Technologies for Plant Pathogen Detection

Biosensors have appeared as advanced detection tools used in many research fields including environmental monitoring, detection of airborne pathogens, real-time detection of human blood components and pathogens and pesticide residues in foods and beverages (Liu et al., 2018). In this review, we refer to a biosensor as a diagnostics device, which typically integrates a biological sensing element and physicochemical transducer to generate an electronic signal when it contacts with a specific analyte of interest or pathogen in solution. Subsequently, a transducer converts a biomolecular interaction into a digital output (Elmer and White, 2018). The biological element that plays the role of a bioreceptor can be antibody, DNA, enzyme, tissue type, whole cell etc. These bioreceptors are responsible to provide recognition specificity to the biosensor through the selective nature of the biochemical interaction. Based on the transducer type, a biosensor may be classified as an electrochemical, optical, thermal, or piezoelectric biosensor (Sawant, 2017).

Last decades have proved that biosensing techniques for plant pathogen detection are practicable to achieve significant diagnostic results through real life applications. Regiart et al. developed a microfluidic electrochemical immunosensor for the early detection of *Xanthomonas arboricola* in walnut plant samples (Regiart et al., 2017a). This *in-situ* diagnostic was three times faster than ELISA and provided significantly higher specificity and sensitivity. Meanwhile, a DNA-probe based bioassay was used by Malecka et al. for Plum Pox Virus (PPV) detection (Malecka et al., 2014). For this, an ion-channel electrochemical biosensor was developed to monitor the interaction between single stranded PPV DNA probes on a glassy carbon electrode surface with a detection limit of 12.8 pg of PPV ssDNA/mL An electrochemical immunosensor was also developed with the same sensitivity to detect PPV virus using an anti-PPV polyclonal antibody attached to gold electrodes (Jarocka et al., 2011).

For the detection of plant pathogens *in-field*, these biosensors are low cost, require little expertize and detect target pathogens very fast. Furthermore, the biosensors are highly specific and sensitive. For instance, detection of *Pseudomonus syringae* with a nanoparticle electrochemical biosensor was more sensitive than conventional PCR, able to diagnose infected plants before any symptoms of the disease appeared (Lau et al., 2017).

### Electrochemical Biosensors

An electrochemical biosensor relies on two core components; a molecular recognition layer and an electrochemical transducer, which converts biological information that is derived from a binding event into an electrical signal that is subsequently shown on a readout device (Ronkainen et al., 2010). In other words, following the active interaction between the analyte and bio-recognition element, a signal generated on the electrode surface is transformed into an electrical signal for quantitative analysis. This class of biosensor is able to detect target pathogens under different conditions including in air, water, and on seeds within different platforms such as greenhouses, *in-field* and in postharvest storage vessels (Fang and Ramasamy, 2015). Among all the possible biological sensing components linked to a transducer, plant’s antibody and DNA are more advantageous and applied in point-of-care assays to detect plant pathogens.

**FIGURE 1:**
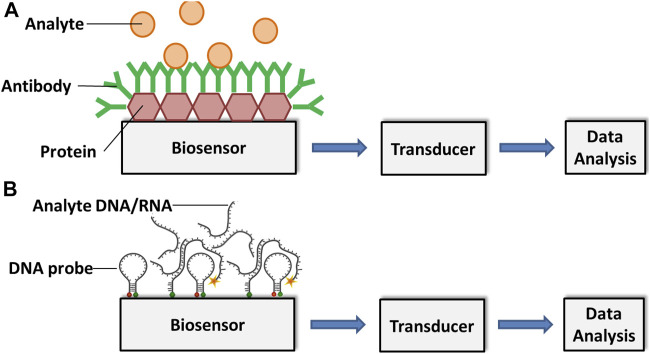
Schematic representation of an **(A)** antibody-based and a **(B)** DNA/RNA-based biosensor for analyte detection. Adapted with permission from Fang and Ramasamy (Fang and Ramasamy, 2015).

Cassedy et al. highlighted the most desirable characteristics of antibodies as their high sensitivity and specificity. They are able to detect only the target antigens at very low concentrations and do not give any signal to antigens other than the interest. Besides, capability to show high affinity level and less or no interactions with other reagents during tests are very important for the effective operation of a biosensor (Cassedy et al., 2020). Again, the fundamental principle of antibody-based immunosensors is the biomolecular interaction between analyte and antibody, which is formed on the transducer surface (Shiddiky et al., 2012a; Shiddiky et al., 2012b) (Figure 1).

Meanwhile, the main principle of DNA-based biosensors relies on hybridization or hydrogen bonding between a target DNA sequence and a DNA probe sequence that is immobilized on a sensing platform. A DNA probe is a fragment of DNA that comprises a nucleotide sequence specific for a chromosomal region of interest (Stratakis, 2008).

**FIGURE 2:**
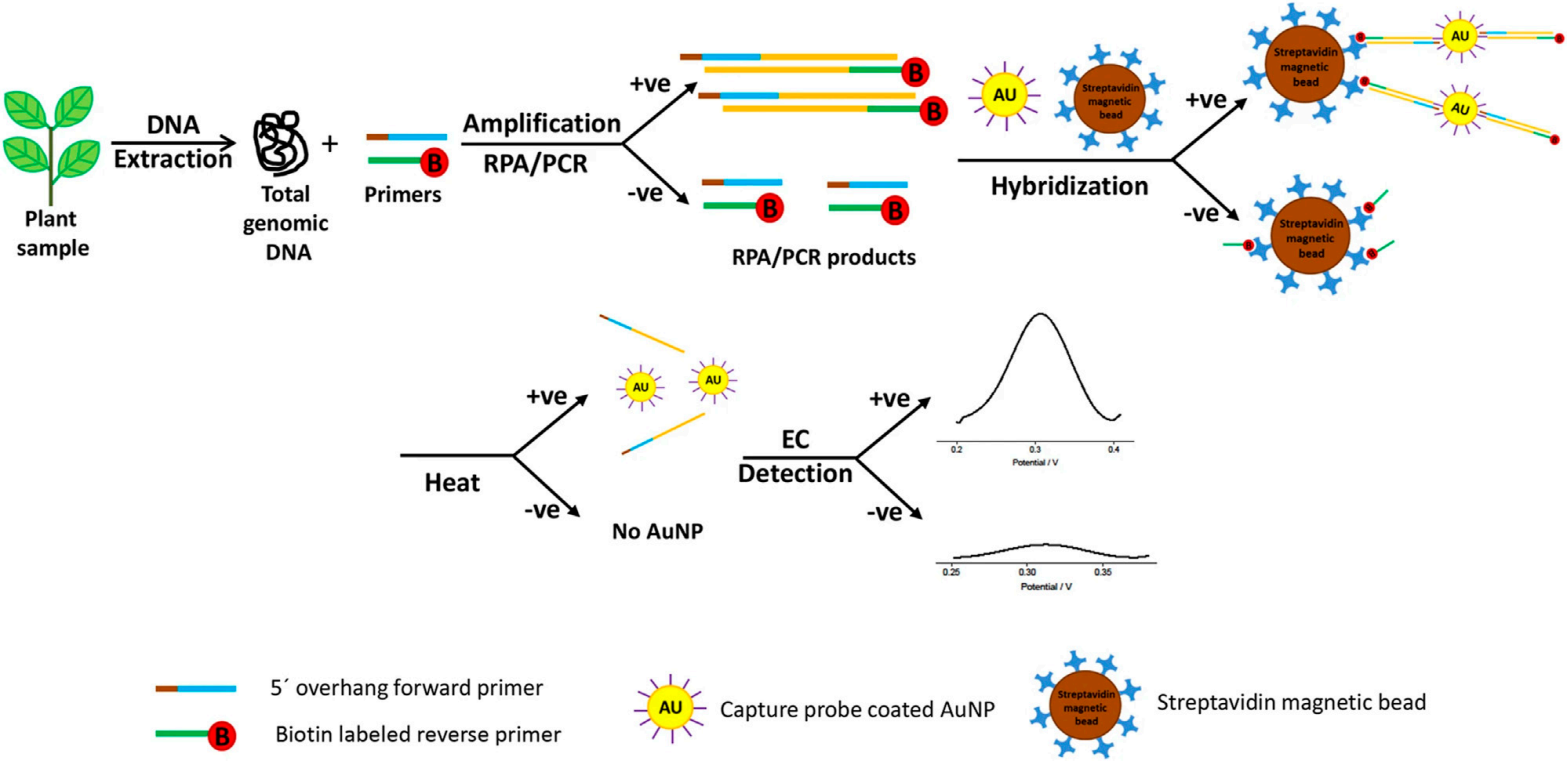
Schematic exemplum of the DNA-based electrochemical bioassay for plant pathogen detection. Adapted with permission from Lau et al. (Lau et al., 2017). EC stands for electrochemical detection and AuNP for gold nanoparticles.

While it is possible to quantify the abundance of pathogens, even down to a single cell, using DNA-based biosensors, DNA rapidly degrades in the environment reducing its sensitivity (Xu et al., 2009). Therefore, methods to improve the sensitivity of this class of biosensor have included development of nano-structured materials with excellent chemical or electronic characteristics to enrich the target sequences and to amplify the detected signal. These have mostly comprised gold, cadmium sulfide or silver nanoparticles with refined biological and chemical properties. These act as substrates for DNA attachment on the sensing surface, increasing the amount of immobilized DNA as well as fulfilling the role of signal amplifiers, dramatically improving accuracy, sensitivity, and the speed of diagnosis. The hybridization process between the target DNA sequence and the DNA probe is defined by the detection of a specific electroactive indicator or the detection of a signal obtained by the most electroactive DNA base (Asal et al., 2018) (Figure 2). DNA-based electrochemical sensors offer an enormous opportunity for on-site environmental monitoring, especially for plant pathogen detection.

According to many authors and recently conducted researches, among various electrochemical biosensors for plant pathogen detection, electrochemical impedance spectroscopy (EIS), quartz crystal microbalance-based (QCM) and voltammetric approaches have been often practiced in laboratories. Here, we review the first two methods which can be employed for some widespread plant pathogens that have continuously damaged worldwide agricultural production.

EIS’s working principle is based on the measure of the opposition to the flow of the current that results from redox reactions and molecular interactions at the electrode surface when voltage is applied to the cell membrane (Shoute et al., 2018). Khater et al. recently developed a labelless impedimetric biosensor for the detect the nucleic acid of citrus tristeza virus and the experiment demonstrated that this method of a high potential can be used in fields to detect plant pathogens. The team obtained the sensing platform using gold nanoparticles distributed on the working carbon electrode where thiolated ssDNA layer and its hybridization with target ssDNA was obtained by faradic impedance measurements. The results illustrated that this DNA sensor’s logarithm relation in the range of 0.1e10 mM of citrus tristeza virus-related DNA with a detection limit of 100 nM with totally 65 min of assay time, 60 min for DNA hybridization and 5 min to read the final electrochemical signal (Khater et al., 2019). Another recent assay was conducted by the team of Cebula et al. who presented label-free EIS to detect *Pseudomonas syringae p*v. lachrymans bacteria on antibody-modified gold electrodes. The team detected Psl with a linear detection range 10^3^–1.2 × 10^5^ CFU/ml (*R* ^2^ = 0.992) and a detection limit of 337 CFU/ml. The assay was 30 times more sensitive than the conventional LAMP method and the detection time took 10 min (Cebula et al., 2019). If we compare these two ligands used in EIS technique, DNA-based biosensors offer better advantages than antibody-based. There are some enhancements that still need to be done with the immunosensor to make them more reliable, specific and sensitive to use in real in-field cases. One of those issues is that whether the selected antigens are able to perform sufficient immune response to generate and separate the pathogen specific antibodies. This emphasizes the importance of antigen used for an assay. Furthermore, as many authors have criticized about utilization of antibodies in sensors, purity of a sample is also vital as there may occur useless interactions with other substances instead of with the target analyte. This factor, in turn, can lead to falsely registered analyte concentration. On the other hand, Menon et al. approved that impedance immunosensors are more sensitive than DNA-based and explained it with the repulsion result between the redox probe $\text{Fe}{\left(\text{CN}\right)}_{6}^{4-}/\text{Fe}{\left(\text{CN}\right)}_{6}^{3-}$ and the negative sugar phosphate backbone of DNA that may impact the final EIS signals. Also, the correct operation of the biosensor needs lots of other factors such as pH, temperature, concentration of ions and length of probe immobilization (Menon et al., 2020).

Most label-free impedimetric biosensors use a self-assembled monolayer to immobilize antibodies on the electrodes. According to Love et al., improved sensitivity is achieved through the use of thiols for self-assembled monolayer formation on gold electrodes (Love et al., 2005). A thiol surface immobilizes protein very efficiently (Lee et al., 2005). This method detected 10 pg/ml of PPV in a titration of just 0.01% of infected plant extract, Which is far more sensitive than the commercially available lateral flow immunochromatographic assay AgriStrip™ (Jarocka et al., 2011).

Meanwhile, a new QCM technology is a label-free and acoustic sensing system which has a remarkable capability for real-time monitoring at the surface of a recognition element within a biosensor (Chen et al., 2018a). QCM sensors consist of a quartz layer installed between working electrodes that oscillates at a resonance frequency generated by the change in mass due to surface binding of the target analyte and biorecognition molecules (antibodies or nucleic acids) (Thies et al., 2017). One important advantage of the QCM biosensor is that it can measure very small mass changes in real time, meaning that it is incredibly sensitive (Şeker and Elçin, 2012). Based on the antigen-binding principle, immunosensors have been developed through the immobilization of antibodies on the QCM surface. However, the common problem with antibodies when they are used as biorecognition material is their fragile structures that make the immobilization process complicated. Poor sensitivity of antibodies to certain physical and chemical conditions like pH, temperature and ionic strength may easily destruct their biological activity and limit the function of the sensor. Although this weak point has been resolved through different methods for immobilizing antibodies including self-assembled monolayers and Protein A linkers which serve to generate a special layer to keep the bioactivity of antibodies making QCM-based immunosensors’ work feasible (Lim et al., 2020). In spite of these improvements, the main consideration of limited lifetime of antibodies will still need further researches because QCM-based immunosensors will not always be reusable and specific detection assays at this position. Another alternative bioreceptor that has been frequently used in QCM-based biosensors is DNA probes. The principle here is based on the immobilized ssDNA probes sequencing with the analyte gene of the pathogen at the crystal surface and obtained frequency response. Unfortunately, many recent researchers prove that DNA performs similar vulnerable characteristics to antibodies related to low stability to surrounding mediums. Modern developments took place last years and new biorecognition elements have appeared which are known as aptamers, single-stranded nucleic acids (DNA or RNA) or peptides with a molecular weight below 25 kDa. Unlike antibodies, aptamers are highly specific to their target molecules like antigens, proteins and nucleic acids with dissociation constants comparable to monoclonal antibodies in the pM range and they present considerable stability to various range of temperatures (Ragavan et al., 2018). Lim et al. analyzed another notable disadvantage of nucleic acid based sensors which lies under incapability to detect the target analyte from a whole cell with genetic material and consequently the nucleic acids need to be separated from the cell before the detection. Then the extracted nucleic acids have to be amplified via expensive PCR method in laboratory to receive enough concentration of analyte that can be detected by the QCM-based biosensor (Lim et al., 2020). Since the main aim is to use the biosensors in point-of-care applications, DNA-based QCM assays also need relevant investigations to acquire highly specific probes and shorten the diagnostic time for pathogens.

Most DNA-based electrochemical biosensors for plant pathogen diagnostics involve either label-free or label-dependent voltammetric detection of DNA hybridization (Khater et al., 2017). The earliest published direct electrochemistry using a DNA method was based on oxidation on a Mercury electrode (Paleček, 1960). The coupling of DNA-based analyte capture systems with electrochemical techniques to measure the quantity of capture has been included in the development of fungal plant pathogen diagnostics tools (Drummond et al., 2003; Privett et al., 2010).

Wang and Li developed the microfluidic microarray assembly method to simultaneously detect three fungal plant pathogens (*Botrytis cinerea*, *Didymella bryoniae*, and *Botrytis squamosa*) (Wang and Li, 2007). They used a glass chip with probe line arrays coupled with a polydimethylsiloxane. The sample flowed through microchannels, crossed the microarray line, and led to rapid DNA hybridization and detection. The advantages of this method included flexible DNA probe development and fast DNA hybridization with small amounts of a sample required (Wang and Li, 2007). Lau et al. successfully developed another highly sensitive technique for plant pathogen DNA detection with the help of colloidal gold nanoparticles as capture probes (Lau et al., 2017). This biosensor involved the amplification of target DNA sequence using recombinase polymerase amplification and hybridization to gold nanoparticle-DNA tags, and was used to identify *Pseudomonas syringae* in infected plant samples (Figure 2). Several examples of existing electrochemical biosensors for plant pathogen diagnostics summarized in Table 1.

**TABLE 1:** Examples of electrochemical biosensors developed for the detection of plant pathogens.

Bio-recognition element	Technique	Crop	Pathogen	Detection limit^a^	Ref
Antibody	Electrochemical impedance spectroscopy-based detection	Plum and tobacco	*Plum pox virus*	10 pg/ml	Jarocka et al. (2011)
		—	*Pseudomonas syringae pv. lachrymans*	2.6 pg/ml	Cebula et al. (2019)
			*Candidatus Phytoplasma*	1.5 × 10^3^ pg/ml	Ebrahimi et al. (2019)
			*Aurantifolia*		
	Quartz crystal microbalance-based detection	Maize	*Maize chlorotic mottle virus*	2.5 × 10^5^ pg/ml	Huang et al. (2014)
	Microfluidic immunosensor	Walnut	*Xanthomonas arboricola*	0.8 pg/ml	Regiart et al. (2017b)
DNA	DNA hybridization voltammetric detection	—	*Plum pox virus*	12.8 pg/ml	Malecka et al. (2014)
		Sugarcane	The sugarcane white leaf (SCWL) disease	4.7 pg/ml	Wongkaew and Poosittisak (2014)
		Cacao	*Phytophthora palmivora*	0.3 pg/ml	Franco et al. (2019)
	Label-free impedimetric method employing gold nanoparticles—modified SPCE	Citrus	*Citrus tristeza virus*	126–1.26 × 10^3^ pg/ml	Khater et al. (2019)

^a^ The limits of detection were converted to the same unit of measurement to be comparable.

### Optical Biosensors

Optical biosensors measure the interaction between a target analyte and ligand using a light source, an optical transmission medium, an immobilized biorecognition element, and a signal detection system. Ultimately, change in amplitude, phase, and frequency of the given light in response to physicochemical conversion (change) generated by the biorecognition process is measured (Ray et al., 2017). Among optical biosensors developed for plant pathogen detection, colorimetric biosensors, fluorescence-based assays-, and surface plasmon resonance-based biosensors are the most common.

Colorimetric biosensors are probably the most wide spread tools that allow the user immediate detection of pathogenic microorganisms in the small number of samples just within 10–15 min via a color change. This type of sensors is widely available in the market. There are two types of this assay: flat-based and solution-based. A lateral flow assay is a flat format of colorimetric tools, a paper-based sensor which is very affordable, easy to use and widely used in laboratories for rapid diagnosis. This analytical tool involves four different pads: The first is made of cellulose where the sample containing the analyte is dropped; the second, pad comprises a glass fiber soaked with a bioconjugate solution; and the third, is the detection or absorption pad where a test line and a control line are printed (Khater et al., 2017). Currently, colloidal gold is most commonly used as a label in commercial lateral flow immunoassays as it has a saturated color and does not require any further method for visualization (Koczula and Gallotta, 2016).

Like the lateral flow assay, the solution based colorimetric sensor operates using the receptor fixed to colloidal gold nanoparticles which reacts to the target pathogens and nanoparticle aggregation results in a color change from red to purple (Yoo and Lee, 2016).

Lateral flow immunoassays, based on colloidal gold nanoparticles, have been developed for several plant pathogens including for Potato Virus X in potato (Drygin et al., 2012), *Fusarium* species in maize (Xu et al., 2019) and *Pantoea stewartii* subsp. *stewartii* (Pss) bacteria in maize (Zhang et al., 2014; Feng et al., 2015). Zhan et al. developed gold nanoparticle-based lateral flow biosensor to detect Phytophthora infestans, the causal agent of late blight in potatoes and tomatoes using a combined integrated universal primer-mediated asymmetric PCR with gold nanoparticle-based lateral flow biosensor. DNA was directly extracted from late blight-infected potato field samples and then was conducted asymmetric PCR amplification and biosensor assay, where the low detection limit of 0.1 pg ml_1 genomic DNA from Phytophthora infestans was obtained with high specificity within 1.5 h (Zhan et al., 2018).

Advantages of this approach include assay simplicity, is quick to provide a result, has a small sample requirement, and has an immediate “point-of-care” diagnosis. However, the lateral flow immunoassays are not as accurate as other nanotechnology-based techniques due to the range of possible inorganic-biological issues leading to possible non-specific adsorption and destruction of target analytes (De Puig et al., 2017). One important drawback of all lateral flow assay-based biosensors is that they lack high sensitivity (Pöhlmann et al., 2014). Many authors find magnetic beads beneficial as signal amplification methods for this limitation. The original property of magnetic beads helps to isolate target cells from complex samples while enabling them to be concentrated by resuspending them in any planned assay volume. Besides, low sensitivity of lateral flow test strips can be resolved with the application of chemiluminescent substrate, multiwell plate and quantum dots instead of colloidal gold (Yoo and Lee, 2016).

**FIGURE 3:**
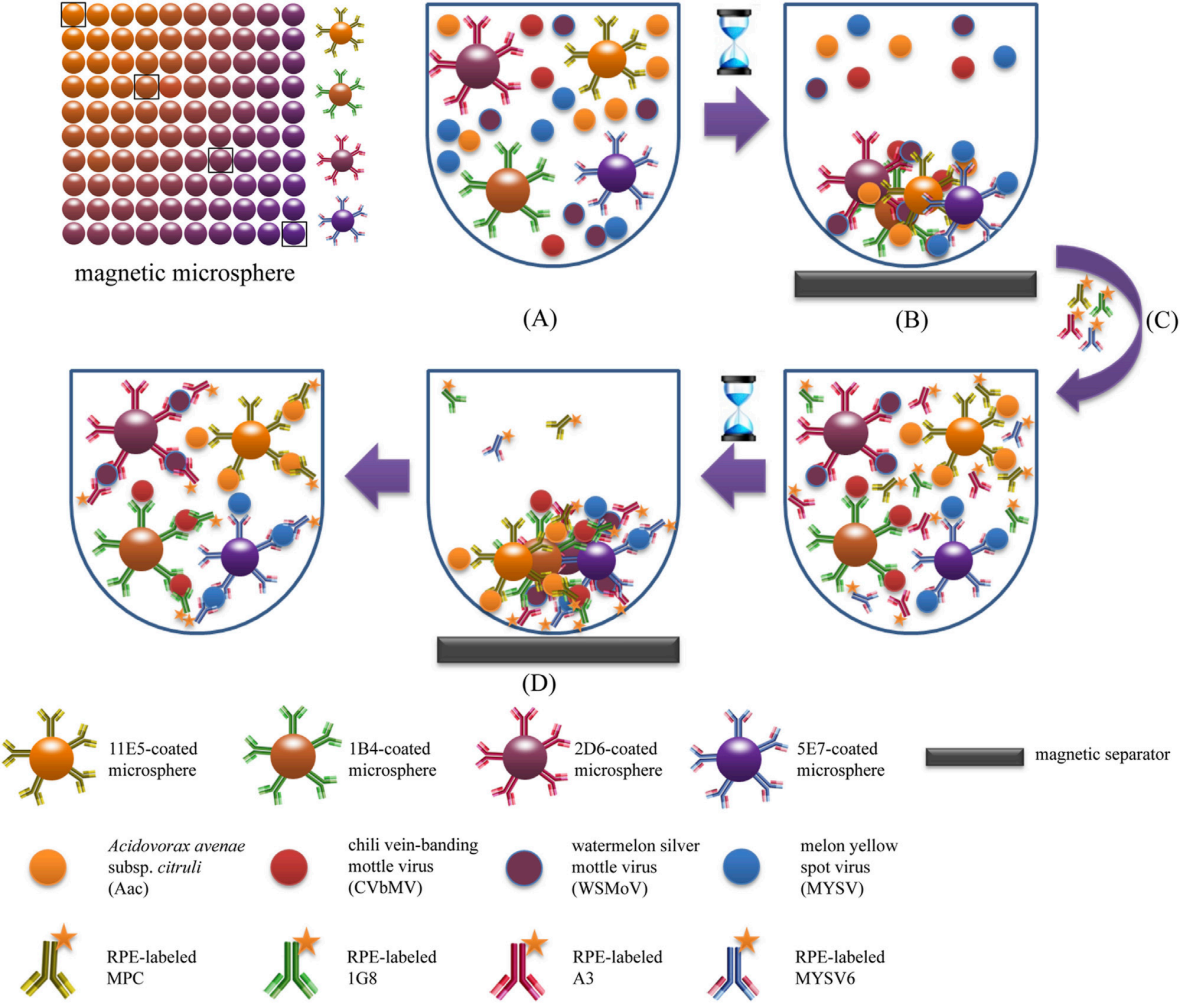
Schematic illustration of magnetic microsphere immunoassay. Adapted with permission from Charlermroj et al. (Charlermroj et al., 2013).

The fundamental principle of fluorescence-based immunoassays relies on the target molecules or antibodies, which are labeled with fluorophores or fluorochrome molecules, producing light during the biological recognition process. Charlermroj et al. reported a novel multiplex detection method, based on a microsphere immunoassay, to simultaneously detect four important plant pathogens: a fruit blotch bacterium Acidovorax avenae subsp. citrulli (Tarasov et al., 2015), Chili Vein-banding Mottle Virus (potyvirus), Watermelon Silver Mottle Virus (tospovirus serogroup IV), and Melon Yellow Spot Virus (tospovirus) (Charlermroj et al., 2013). The working principle of this method was based on using antibodies coupled with fluorescence-coded magnetic microspheres to capture the pathogen of interest. Next, the pathogen presence was determined by R-phycoerythrin-labeled antibodies with an assay time of just 1 h (Charlermroj et al., 2013) (Figure 3). Despite the possible highly sensitive detection, all immunoassay-based methods suffer from limitations. As previously outlined for traditional immunoassays, this includes the requirement for preparation of monoclonal antibodies (Skottrup et al., 2008), and the dependency on the sample and/or environment in which the assay is conducted in potential for cross-reactivity of antibodies with endogenous and exogenous substances, producing false negative results or reduced sensitivity (Dodig, 2009).

Lternatively, surface plasmon resonance-based biosensors are predominantly used in optical biosensing techniques with the advantages of label-free, real-time and highly accurate detection (Homola, 2008; Carrascosa et al., 2014; Sina et al., 2014; Sina et al., 2016). The devices contain a sensor chip that is a surface constructed of a metal, such as gold, within two layers comprised of glass and a liquid. The analyte flows over the surface of the chip entering through the bottom or liquid layer and binds with the immobilized ligand that illuminates a light signal that is detectable at a specific angle. The generated signal is then observed with a surface plasmon resonance sensorgram (Damborský et al., 2016). Lin et al. developed a label-free surface plasmon resonance biosensor based on gold nanorods for detection of two prevalent and economically important orchid viruses: Cymbidium Mosaic Virus and Odontoglossum Ringspot Virus (Lin et al., 2014).

Recent progress made in nanotechnology provide a wide range of potential nanomaterials. According to Li et al. gold nanoparticles, with a diameter of 1–100 nm present optimal surface-to-volume ratios and energies to support the immobilization stability for a large range and quantity of biomolecules without changing their bioactivity (Li et al., 2010). Also, gold nanoparticles have high levels of electron conductivity. Consequently, their use has greatly impacted on the reliability, sensitivity and rapidity of both optical and electrical biosensors (Li et al., 2010). For diagnosis of plant pathogens, many lateral flow assays based on DNA hybridization to gold nanoparticles have been developed including for *Acidovorax avenae* subsp. *citrulli* (Tarasov et al., 2015) of watermelon (Zhao et al., 2011) and many others summarized in Tables 1 Tables 2. This is currently a fast evolving approach and was has huge potential for the pre-emptive diagnostic-guided intervention in informed disease management, however, several limitations and considerations, as outlined below.

**TABLE 2:** Examples of optical biosensors developed for the detection of plant pathogens.

Bio-recognition element	Technique	Crop	Pathogen	Detection limit^a^	Ref
Antibody	Lateral flow immunoassay	Potato	*Potato virus x*	2 × 10^3^ pg/ml	Drygin et al. (2012)
		Maize	*Pantoea stewartii sbusp. stewartii*	538 pg/ml	Feng et al. (2015)
		Maize	*Pantoea stewartii sbusp. Stewartii*	5.38 pg/ml	Zhang et al. (2014)
	Surface plasmon resonance immunoassay	Orchid	*Cymbidium mosaic virus*	48 pg/ml	Lin et al. (2014)
			*Odontoglossum ringspot virus*	42 pg/ml	
	Microsphere immunoassay	Watermelon	*Acidovorax avenae subsp. Citrulli*	3.5 × 10^3^ pg/ml	Charlermroj et al. (2013)
			*Watermelon silver mottle virus*	20.5 × 10^3^ pg/ml	
			*Melon yellow spot virus*	35.3 × 10^3^ pg/ml	
		Datura	*Chilli vein-banding mottle virus*	10^3^ pg/ml	
DNA	Bridging flocculation	—	*Pseudomonas syringae*	0.5 pg/ml	Wee et al. (2015)
	Gold nanoparticles-based colorimetric detection	—	*Pseudomonas syringae*	15 pg/ml	Vaseghi et al. (2013)
	Gold nanoparticles-based lateral flow assays	Banana	*Banana bunchy top virus*	3.2 × 10^4^ pg/ml	Wei et al. (2014)
		Watermeon	*Acidovorax avenae subsp*		
		*Citrulli*		7.25 × 10^4^ pg/ml	Zhao et al. (2011)
		Potato	*Phytophthora infestans*	10^–4^ pg/ml	Zhan et al. (2018)
	Electrochemiluminescence-based DNA analysis	Banana	*Banana bunchy top virus*	8.3 pg/ml	Tang et al. (2007)
			*Banana streak virus*	0.1 pg/ml	
	Microfluidic microarray assembly method	Cucumber	*Botrytis cinerea*	2.7 × 10^4^ pg/ml	Wang and Li (2007)

^a^ The limits of detection were converted to the same unit of measurement to be comparable.

## Biological and Technical Challenges Associated With In-Field Detection and Quantification

The role of routinely practiced conventional methods in plant pathogen detection is quite important and effective. In particular, immunology-based ELISA, nucleic acid-based PCR and LAMP approaches have been implied widely in laboratories and *in-situ* real time diagnostics in the present time. However, they have still outstanding drawbacks that need to be deeply researched to fill those critical gaps. For example, ELISA is an antigen-antibody binding reaction which still has low specificity and due to the complex design of antibodies cross-reactivity may occur and production of relevant bioreceptors (monoclonal antibodies) requires high cost. Similarly, a well-established PCR-based technology is also complicated with the requirements for expensive instruments and costly commercial reagents which consequently may not be suitable for desirable point-of-care detection (Li et al., 2015). Besides, for the nucleic acid-based assays, there are several steps that need to be done to disintegrate the target cells prior to the detection process which in turn require professional laboratory workers (Chen et al., 2018b).

Recent advances in micro- and nanotechnologies has enabled the development of biosensor-based assays that are highly specific, sensitive, and provide rapid results. However, there are many further considerations and potential challenges to consider in their broad adoption and implementation within agricultural and horticultural settings. Biosensors themselves will likely become a common tool within an IDM package but each will need careful validation—potentially through agroeconomic and agroecological modeling—to be applicable within a particular crop, location and/or a pathosystem. Their application for decision support purposes, in terms of disease management, must be done in conjunction with reliable knowledge on the plant phenology, pathogen biology, and the disease epidemiology. Pathogen detection and quantification information must also be used in conjunction with multiple other factors within each farming system. This includes cultural cropping and climatic factors as well as the current disease management practices being undertaken. This may also lead to predictive tools for epidemics and futureproofing of IDM strategies to protect crop productivity and quality. Such validated biosensor tools would greatly improve the accuracy of epidemiology models such as that of Gonzalez-Dominguez et al., for *Botrytis cinerea* on grapevines (Gonzalez-Dominguez et al., 2015).

In addition, the accuracy for biosensor-based detection is largely dependent on the *in-field* sampling strategy. This is inclusive of the sampling design (spatial and temporal), the time it takes to conduct the sampling and the method used to expose the pathogen-associated analyte to the biosensor probe (Ray et al., 2017). As a case in point, the chickpea fungus *Ascochyta rabiei* survives on infected seed and crop residues and the spores can be carried to other crops by wind or rain splash (Davidson and Kimber, 2007). Therefore, an informed sampling method should be adhered to, guided by climatic, host, and farming system knowledge.

Detection limits for target pathogens may be greatly impacted by their biological concentrations on or within plant materials or other environmental samples tested. This may also be affected by abundance of non-target molecules, which interfere with probe binding, reducing the final detectable signal. When nucleic acids are used as target sequences to hybridize to a complementary capture probe the reaction is open to influences on the binding accuracy and specificity within the diagnostic environment, potentially introducing false-positive responses from non-specific capture on the sensing surface. The complexity of the sample matrix that contains ions and cells may disrupt the optimization of amplifiers (Vidic et al., 2019).

*In-field* sample preparation methods remain largely unexplored despite their important role in the overall diagnostics procedure and interpretation (Capote et al., 2012). Indeed, an initial separation of the target analyte from the sample background is an important step to improve detection (Sin et al., 2014). In conventional assays, sample preparation methods like centrifugation and precipitation have been used to solve these issues. However, these techniques are less conducive to *in-field* applications since they require multiple pieces of powered equipment and are time-consuming (Chiu et al., 2010). The majority of PCR-based diagnostics rely on Taq DNA polymerase activity and are dependent on removal of PCR inhibitors during sample preparation, such as polysaccharides and phenolic compounds, often occurring in plant and fungi tissues (Capote et al., 2012). Direct sequencing methods are even more sensitive to impurities in the input material and require high molecular weight pure DNA to produce reliable results. Although there are several commercial kits available for DNA extraction from pathogens, many fail to extract high quality DNA from environments that contain higher concentrations of acids such as humic acid in soil or phenolic acids in plant tissues. To overcome this challenge, modified nucleic acid (DNA or RNA) extraction techniques are required to remove background interference (Islam et al., 2017; Bilkiss et al., 2019). However, these methods are laborious and time-consuming protocols that use liquid nitrogen or dry-ice for sample homogenizing and toxic reagents such as cetyltrimethyl ammonium bromide, phenol/chloroform and β-mercaptoethanol for nucleic acid extraction. Furthermore, different pathogens require different sampling processing steps. For instance, fungi spores are much more complicated than bacteria and contain much more secondary metabolites that interfere with DNA purification, so it requires more effort to extract DNA.

As an alternative, the magnetic properties of metal nanoparticles themselves may be utilized, to separate and concentrate the bound target analytes. The further development of paramagnetic bead-based DNA extraction and purification methods will substantially improve the speed and quality of DNA extractions, while reducing the dependency on toxic reagents and powered centrifuge and heatblocks (Berensmeier, 2006). Lab-made paramagnetic particles reagents (Oberacker et al., 2019) or commercial kits, such as the CleanNA Clean Plant DNA Kit and MagBio HighPrep™ Plant DNA Plus Kit, should be further evaluated for their cost, time and labor-efficiency, and the quality and yield of the extracted DNA. This approach will be especially useful when analyte capture and separation are able to be performed as a twostep reaction in a single reaction tube with subsequent immediate loading onto the biosensor chip.

Timing from sampling to result, is also of immediate concern with optimal times of less than a few minutes per sample *in-field* a necessity for a grower, pathologist, or scientist to sample an entire farm or even region within a manageable timeframe. Recent progress in fast-tracking diagnostics has been made, for example, for *Phytophthora ramorum* and *P. kernoviae* using a lateral flow device with an overall efficiency of 95·6% and with results within 3–5 min (Lane et al., 2007). Another main factor is affordability—massive reduction in cost per sample within nano-biosensor devices has already been achieved using bare screen-printed carbon electrodes ($2.50 USD per test) and streptavidin coated screen-printed carbon electrodes ($5 USD per test) (Lau et al., 2017). The commercial AgriStrip™ and Pocket Diagnostic kits cost <$10 USD per sample (Singh et al., 2020), although these are not quantitative. There remains an immediate need to develop equally sensitive and specific affordable biosensor diagnostic devices that are quantitative and that are able to detect multiple targets in a single assay, for accurate and truly informed disease management decision support.

Despite the advantages of electrochemical and optical biosensing techniques over the conventional methods mentioned in this review, there is a need for further research on implementing this technology in plant pathogen quantification under *in-field* conditions. A validation of a portable sensor requires a specialized hardware, which can be expensive and difficult to operate for un-specialized such as farmers. Currently it is unknown what minimum inoculum levels are required to induce a disease in host plants. Establishing this threshold is essential to translate the biosensor quantified pathogen level to estimate the risk of disease..

## Conclusion

Nano biosensing technologies and devices are fast replacing conventional and traditional diagnostic tools. With further optimization for application in a range of environments, the use and validation of these affordable, fast, and highly sensitive and specific tools for plant pathogen detection in the field will become widely adopted in the near future. It is highly likely that with further validation, these tools will also be used for modelling disease and will therefore become an essential part of a proactive and pre-emptive suit of IDM tools, for use by growers and agronomists ahead of epidemics. Their use will most likely substantially reduce the frequencies and amounts of chemical applications to crops both pre- and post-harvest, as well as reduce on-farm production costs and loss of quality and yield from disease. Further research to refine these nano biosensors will concentrate on multiplexing, to enable simultaneous surveillance for, and detection of, multiple disease-causing pathogens. The ability to wirelessly and remotely capture, sense, and signal pathogenic organism presence, frequency, and quantity above and below ground will be on the cards to further future proof our plant-derived foods.
